# Integrating Point-of-Care Bacterial Fluorescence Imaging-Guided Care with Continued Wound Measurement for Enhanced Wound Area Reduction Monitoring

**DOI:** 10.3390/diagnostics14010002

**Published:** 2023-12-19

**Authors:** Rosemarie Derwin, Declan Patton, Helen Strapp, Zena Moore

**Affiliations:** 1 School of Nursing and Midwifery, Royal College of Surgeons in Ireland (RCSI), University of Medicine and Health Sciences, Dublin D02 YN77, Ireland; 2 Fakeeh College of Health Sciences, Jeddah 23323, Saudi Arabia; 3 Faculty of Science, Medicine and Health, University of Wollongong, Wollongong NSW 2522, Australia; 4 School of Nursing & Midwifery, Griffith University, Gold Coast, QLD 4222, Australia; 5 School of Health Sciences, Faculty of Life and Health Sciences, Ulster University, Belfast BT15 1AP, UK; 6 Department of Nursing, Fakeeh College for Medical Sciences, Jeddah 23323, Saudi Arabia; 7 Department of Public Health, Faculty of Medicine and Health Sciences, Ghent University, 9000 Gent, Belgium; 8 Lida Institute, Shanghai 201609, China

**Keywords:** wound healing, autofluorescence imaging, bacteria, wound care, MolecuLight, debridement

## Abstract

Aim: This prospective observational study investigated wound area reduction (WAR) outcomes in a complex wound population composed of non-healing acute and chronic wounds. The relationship between bacterial autofluorescence signals and WAR was investigated. Area measurements were collected both manually and digitally, and both methods were compared for accuracy. Methods: Twenty-six participants with 27 wounds of varying etiologies were observed twice weekly for two weeks. Digital wound measurement, wound bacterial status assessment, and targeted debridement were performed through a point-of-care fluorescence imaging device (MolecuLight^®^ i: X, MolecuLight Inc, Toronto, Canada). The wound area reduction (WAR) rate was calculated using baseline and last visit measurements. Statistical analyses, including *t*-tests, Fisher exact tests, the Wilcoxon signed rank test for method comparison, and ANOVA for bacterial subgroups, were applied as pertinent. Results: The overall average WAR was −3.80 cm^2^, or a decrease of 46.88% (manual measurement), and −2.62 cm^2^, or a 46.05% decrease (digital measurement via MolecuLight^®^ device). There were no statistically significant differences between the WAR of acute and chronic wounds (*p* = 0.7877). A stepwise correlation between the WAR and bacterial status classification per fluorescence findings was observed, where persistent bacteria resulted in worse WAR outcomes. An overestimation of wound area by manual measurement was 23% on average. Conclusion: Fluorescence imaging signals were linked to WAR outcome and could be considered predictive. Wounds exhibiting bacterial loads that persisted at the end of the study period had worse WAR outcomes, while those for which management was able to effectively remove them demonstrated greater WAR. Manual measurement of the wound area consistently overestimated wound size when compared to digital measurement. However, if performed by the same operator, the overestimation was uniform enough that the WAR was calculated to be close to accurate. Notwithstanding, single wound measurements are likely to result in overestimation.

## 1. Introduction 

Non-healing, complex wounds are a significant problem for both the individual and the health care system alike. A complex wound is a type of wound that does not heal with conventional treatments and/or within a reasonable time frame. These wounds can be either acute or chronic and often result in significant damage to the epidermal and dermal layers of the skin, as well as the underlying subcutaneous tissues [1,2,3]. Recent estimates have reported a point prevalence for wounds between 1.47 and 4.47 per 1000 [4,5,6,7]. However, with the rising aging population compounded with an increasing rate of wound-related comorbidities (e.g., cardiovascular disease, diabetes, and obesity), the healthcare disturbances caused by the COVID-19 pandemic, and persistently uncontrolled rates of surgical site infections and antibiotic resistance, this is set to escalate further [1,8,9]. Complex wounds incur substantial direct and indirect costs, impacting patients’ work, independence, and mental well-being. Studies reveal reduced quality of life, mobility, self-esteem, and increased anxiety and depression in individuals with unhealed wounds. This burden extends to healthcare system expenditures, with chronic wounds costing significantly more than promptly healed wounds in both the UK and the US [4,5,8,9,10,11,12].

All these burdens could be lessened if wound care shifts from a reactive approach towards a proactive and preventative one, where the wound healing process is reinitiated before tissue deterioration is severe and before the onset of infection and its complications. The persistent presence of biofilm and bacteria at pathogenic loads in and around the wound is one of the main obstacles to healing [13,14,15]. Its eradication, irrespective of clinical expression, is essential to wound healing to avoid the downstream complications that impaired wound healing and infection carry. Despite a wealth of evidence supporting this notion, our field has not universally embraced this approach. Clinicians continue to rely on clinical signs and symptoms of wound infection such as pain, erythema, edema, heat, and pus to guide intervention decisions, but these signs may be unreliable in the context of chronic wounds and do not necessarily reflect the bacterial load present [16,17]. The conventional belief that infection and inflammation only occur when bacterial levels exceed 10^5^ CFU per gram of tissue persists. However, studies challenge this, indicating adverse effects on healing even below this threshold [13,15,18]. The concept of “critical colonization” is being redefined as “chronic inhibitory bacterial load” (CIBL) by Armstrong et al. [19], considering that high bacterial loads hinder healing without causing infection. Biofilms, often silently present in chronic wounds, pose challenges due to their clinical invisibility and low detection rates in standard swab samples. Their disturbance through debridement is crucial for positive outcomes, yet most indications for debridement are ironically contingent on the presence of overt clinical inflammation and/or infection [20,21,22].

Traditionally, no point-of-care tool has existed that could overcome this issue and detect bacteria and biofilm, thereby contributing to a lack guidance for care decisions at the bedside. Three common techniques—deep-tissue biopsy, needle aspiration, and swab culture—are used to identify colonization or infection in addition to clinical assessment. Swab culture is favored for its practicality, simplicity, non-invasiveness, and cost-effectiveness [23,24]. However, there are discrepancies between swabs and gold standard biopsy results, as swabs may not detect all microbial types and cannot assess deep-tissue microbial levels. Gardner et al. [25] previously highlighted the inaccuracy of semi-quantitative wound swab cultures compared to quantitative wound biopsy methods. A more recent study concurred with these findings, demonstrating that semi-quantitative cultures produce a highly variable range of bacterial loads within each category [26]. Wound biopsies, while accurate, are less commonly performed due to their invasiveness and patient discomfort. Faced with these limitations, clinicians often rely on subjective, unstandardized criteria based on visual observation of wounds [27,28].

While traditional diagnostic methods offer valuable insights, they are time-consuming and have variable results that are often reliant on clinician experience. The demand for improved, objective bacterial detection and accurate wound assessment tools has led us to employ the MolecuLight^®^ i:X, a real-time, non-invasive point-of-care diagnostic imaging device. This tool facilitates the detection of moderate to heavy bacterial loads in biofilm and planktonic form [29,30] equal to or exceeding 10^4^ CFU/gram of tissue. It leverages bacterial auto-fluorescence principles by illuminating the wound with a safe violet light and it reveals endogenous fluorescence signals from tissues and bacteria, providing real-time bacterial status assessment [16,31,32]. Importantly, its utilization does not necessitate extensive training, and it has been employed in a multitude of care settings with good detection, healing, and predictive outcomes [33,34,35,36]. 

Recognizing the benefits of early intervention and bacterial management is crucial. Achieving a wound area reduction (WAR) of 20–40% or more within the initial 2–4 weeks is a positive prognostic indicator for healing [37,38,39]. For comprehensive patient evaluation, consistent and accurate wound assessment and measurement are essential. Despite the importance of these measurements, existing wound measurement strategies exhibit considerable inter and intra-reader variability, frequently resulting in overestimation—up to 40% [40,41,42]. There is a need for enhancements in both bacteria detection and wound measurement for challenging wounds.

## 2. Materials and Methods

This is a prospective observational study following patients with complex acute and chronic non-healing wounds of varying etiologies over a 2-week period. Reporting was conducted according to the Strengthening of the Reporting of Observational Studies in Epidemiology (STROBE) guidelines [43]. The 10 articles of the Nuremberg Code, the basic ethical principles from the Belmont Report, and the Code of Professional Conduct for Nurses and Midwives [44] were adhered to throughout the study. Ethics approval was sought from the Research Ethics Committee in January 2019 and approved on the 31 July 2019 (Approval number: 2019-06 (02)). The sample size was estimated based on the findings from a previous study by Derwin et al. [45].

All participants provided informed consent after having received both written and verbal study information. The researcher confirmed their understanding before seeking consent. The patient information leaflet, assessed for readability, was given at least 24 h in advance. Dignity, privacy, and data confidentiality were maintained. All data, including MolecuLight^®^ i:X images, were securely stored on a password-encrypted computer.

### 2.1. Aims

There were three aims of this study. First, to determine the wound area reduction (WAR) rate for complex wounds at a specialized wound care center utilizing fluorescence imaging to guide their treatments. Secondly, to establish the relationship between bacterial fluorescence imaging (MolecuLight^®^ i:X) findings and wound healing as depicted by the WAR from the baseline to study’s end in a variety of wound types. The third aim was to compare the ruler (manual) measurements of the wounds and the digital measurements as captured by the MolecuLight device to assess for any variability in single time point measurements as well as in the final WAR calculation (change over time).

### 2.2. Inclusion and Exclusion Criteria

Consecutively assessed patients with non-healing wounds were included in the study. Non-healing wounds were defined as those showing no signs of healing and closure over a two-week period. Eligible patients for inclusion were those attending the dressing clinic at least twice weekly and receiving treatment for a minimum of 2 weeks, provided they consented to participate. Exclusion criteria consisted of patients who would not regularly attend the wound clinic and those who did not give their consent.

### 2.3. Data Collection

Clinical staff screened participants for medical purposes only; an independent researcher (RD) collected data for research purposes in an independent, password-protected database, including name, age, gender, smoking status, co-morbidities, wound etiology, and duration. Data collection occurred at baseline and twice weekly (every 4 days) for two weeks, aligning with the need for frequent wound healing evaluation [46,47].

### 2.4. Patient Assessment and Care Protocol

This study adopted the principles of TIME and Wound Bed Preparation [20,48] performed by a nurse in the dressing clinic: (1) Cleanse—using saline; (2) Debride—using curettage; the extension, intensity, and location of the debridement was influenced by the fluorescence findings noted at the bedside. Fluorescence signals were targeted via cleansing, debridement, and topical antibiotic/antiseptic management. (3) Manage—using Iodine (Iodoflex^®^) and gauze dressing or a foam dressing depending on the level of exudate when appropriate; (4) Dressings changed twice weekly for 2 weeks. Manual and digital wound measurement and fluorescence imaging were performed after step 1 (cleansing).

### 2.5. Manual Wound Measurement

At each visit, a ruler was used to measure wound length (longest axis) and width (greatest perpendicular axis), from which the area was calculated by multiplying the length by the width. The depth and any undermining were measured using a wound measuring stick at each study visit.

### 2.6. Digital Wound Measurement and Fluorescence Imaging

A handheld fluorescence imaging device (MolecuLight^®^ i:X, MolecuLight Inc., Toronto, ON, Canada) was used for digital wound measurement and bacterial assessment, validated for accuracy and safety in previous studies. Operating without patient contact or contrast agents, it detects bacterial autofluorescence using safe violet light, indicating bacterial presence up to 1 mm depth [29,32].

**Figure 1:**
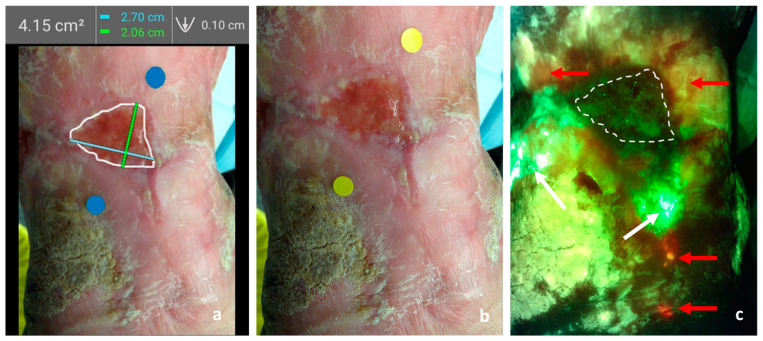
Sample images obtained through the MolecuLight *i*:X device and used for digital measurement and bacterial autofluorescence assessment. (**a**) Digital measurement performed over the standard image of the wound. Area, length, and width are automatically provided by the software, while depth is manually inputted. (**b**) Standard image of the wound. (**c**) Fluorescence image of the wound. The interrupted line indicates the outline of the wound for easy reference, surrounding it there are cyan fluorescence signals corresponding to Pseudomonas (white arrows) and other gram +/− (red arrows) bacteria at loads greater than 10^4^ CFU/gr of tissue. Note that what is referred to as red fluorescence can vary in hue from bright red to pink or orange. This phenomenon stems from the overlapping of normal tissues with the red signals of bacterial autofluorescence.

The independent researcher (RD) captured images after dressing removal, cleansing with saline (which helps prevent visual contamination from confounding substances such as products containing silver or iodine that may block/absorb fluorescence light), and a 30 min acclimatization period. Following device guidelines, two yellow stickers marked wound boundaries for automatic estimation, placed 8 cm to 12 cm from the wound. The device measured width and length, automatically calculating, and saving the area data over a standard (non-fluorescence image Figure 1a). Standard and fluorescence images were obtained, with darkness confirmed/achieved using an ambient light sensor and/or DarkDrape^®^.

Interpretation of the fluorescence images was performed by a trained researcher (RD) on the subject. In short, cyan, and red signals visualized on the device are predictive of potentially pathogenic bacteria at moderate to heavy bacterial loads. Red fluorescence signals indicate the presence of aerobes/anaerobes, Gram-positive/negative bacteria (>10^4^ CFU/g of tissue) including *Staphylococcus* aureus, *E. coli*, and *Proteus*, among many others. A blush red or yellow fluorescence signal suggests the presence of sub-surface bacteria. Finally, cyan fluorescence signals indicate the presence of Pseudomonas aeruginosa [16,31,32]. Figure 1 depicts sample images obtained through the MolecuLight i:X device and used for digital measurement and bacterial autofluorescence assessment.

### 2.7. Statistical Analysis

Statistical analysis of the participants’ demographic and clinical data was performed using STATA (version 15.1) and GraphPad (Version 10.0.2). Analyses were conducted in the larger cohort (*n* = 27 wounds) and for two distinct cohorts based on wound duration: acute and chronic wounds. The cut-off to determine acute versus chronic was established at 6 weeks or 42 days. The acute wound cohort had an n of 16, whilst in the chronic wound cohort 11 wounds were included for analysis. 

Appropriate descriptive and inferential analyses were conducted. Descriptive statistics included means, standard deviations, counts, and percentages. Frequency distributions described categorical data (e.g., gender, comorbidities, wound type, and location of bacterial burden), while means and standard deviations characterized continuous data (e.g., age, duration of the wound, and wound size). The wound reduction rate was calculated as follows: wound reduction rate (%) = (baseline area week 1 day 1, area at week 2 day 4)/baseline area × 100 [49,50]. When appropriate, unpaired t-tests and Fisher exact tests were applied. Statistical significance for WAR as measured with the two methods (ruler and digital) was calculated using the Wilcoxon matched pairs signed rank test. ANOVA was employed to determine the statistical significance amongst bacterial classification subgroup WAR results.

## 3. Results

### 3.1. Description of the Study Population

A total of 27 wounds across 26 patients were included in the analysis, just over half of which were acute in nature (present for <6 weeks at admission). The patients’ mean age was 47 years (SD ±20.3, range 18–81) for the entire study group, and this was not significantly different between the acute and chronic wound cohorts (*p* = 0.44, unpaired *t*-test). Most patients were male (79%), and similarly, sex did not differ based on wound chronicity (*p* = 0.34, Fisher’s exact test). Most patients in both cohorts had at least one comorbidity that would impact wound healing, and more patients with chronic wounds were current or former smokers. There were some differences in wound location between the acute and chronic cohorts; however, for the most part, wounds occurred on the lower body (foot, buttocks, abdomen, lower leg). The mean duration of the participants’ wounds was 53.88 days (SD: ±64.49, range 3–217 days). Table 1 outlines the demographic and wound characteristics among these patients.

### 3.2. Wound Area Reduction (WAR)

Wound area reduction (WAR) values were determined for each of the 26 wounds by comparing the recorded surface area measurements at visits 1 and 4, there was a maximum time gap of 2 weeks between them. Both manual and digital measurements were considered during this analysis.

The WAR values differed slightly depending on the measurement method used, where the average WAR among all wounds was −3.80 cm^2^, or a percentage decrease of 46.88%, when measuring manually (via ruler), versus −2.62 cm^2^, or a 46.05% decrease, when measuring using the digital feature of the MolecuLight^®^ device. These differences in WAR due to measurement technique were statistically insignificant both in terms of percentage change (*p* = 0.9057, Wilcoxon matched-pairs signed rank test) and WAR as quantified in cm^2^ (*p* = 0.2017, Wilcoxon matched-pairs signed rank test).

Of note, the WAR was nil (no change) or positive (increased area) for four wounds when measuring manually, but only for three wounds when measuring digitally. When comparing the WAR results for the acute versus chronic wound cohorts, there were no statistically significant differences in cm^2^ WAR (*p* = 0.7877, Kolmogorov–Smirnov test) or percentage change (*p* = 0.9662, unpaired t-test). Table 2 depicts in detail the WAR of the entire cohort and provides a breakdown per wound duration (chronic and acute).

The bacterial status of the wound, as determined by the presence or absence of fluorescence signals both at the outset of treatment and at the study’s final visit, seemed to influence the WAR observed (Table 3). To enable this analysis, we created the following bacterial status classifications: *pos-pos* (positive at visit 1, still positive at visit 4), *pos-neg* (positive at visit 1, negative at visit 4), *neg-pos* (negative at visit 1, positive at visit 4), and *neg-neg* (negative at visit 1, still negative at visit 4). As only two wounds were neg-pos (both acute), we were unable to subdivide further based on wound duration (acute/chronic). Thus, the results below correspond to the entire cohort.

**Figure 2:**
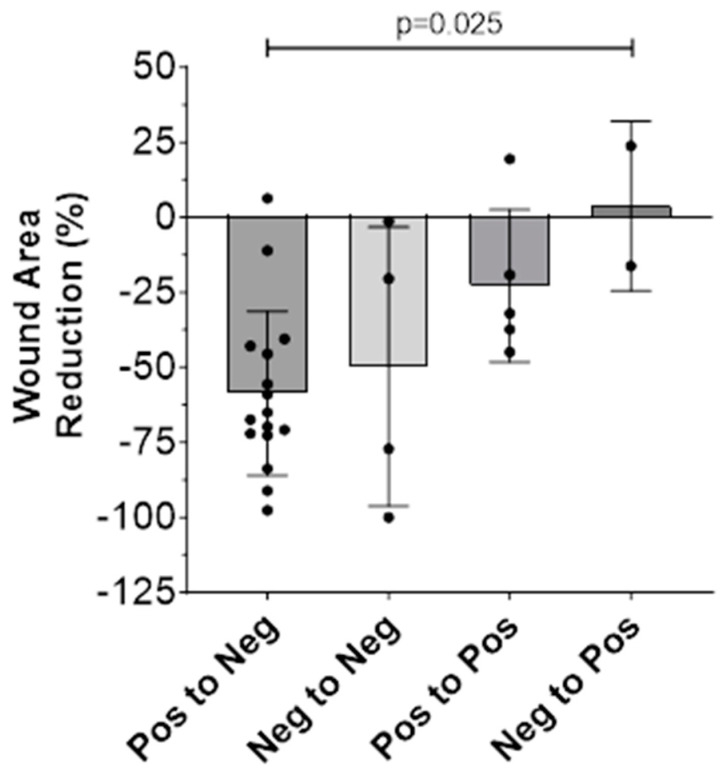
Percentage wound area reduction (WAR) as measured digitally using the MolecuLight device and subdivided based on wound bacterial status (positive or negative) at the beginning (visit 1) versus the end (visit 4) of the study period. A “pos” (i.e., positive) classification denotes the presence of red and/or cyan bacterial fluorescence, indicative of most Gram-positive, Gram-negative, aerobic, and anaerobic bacterial species along with *Pseudomonas* aeruginosa at loads > 10^4^ CFU/g. A “neg” (i.e., negative) classification denotes the absence of bacterial fluorescence.

We observed a stepwise correlation between the WAR and bacterial status classification (Figure 2), where there was a statistically significant difference in mean percentage WAR among the four classifications (*p* = 0.025, F = 3.735, ordinary one-way ANOVA). Respectively, the mean WAR percentage was −58.63% for pos-neg, −49.70% for neg-neg, −22.80% for pos-pos, and +3.80% for neg-pos. In essence, wounds exhibiting initial bacterial loads that were resolved by the final visit exhibited the most favorable outcomes, followed by those initially negative and remaining so until the final visit. Wounds with persistent bacterial loads from the outset to the final visit demonstrated less favorable outcomes. Finally, wounds that started as negative but accumulated bacterial loads by the final visit fared the worst, with a positive percentage WAR indicating an increase in average wound area. It should be noted that only two wounds were classified as neg-pos, and therefore this result should be interpreted with caution.

### 3.3. Wound Measurement Manual (Ruler) versus Digital (MolecuLight i:X)

Complete wound measurement data were obtained for all 27 wounds under investigation. However, one wound had fully healed by week 4, making further measurements unattainable and registering as “0”. This resulted in a total of 107 unique data points for each measurement method.

**Figure 3:**
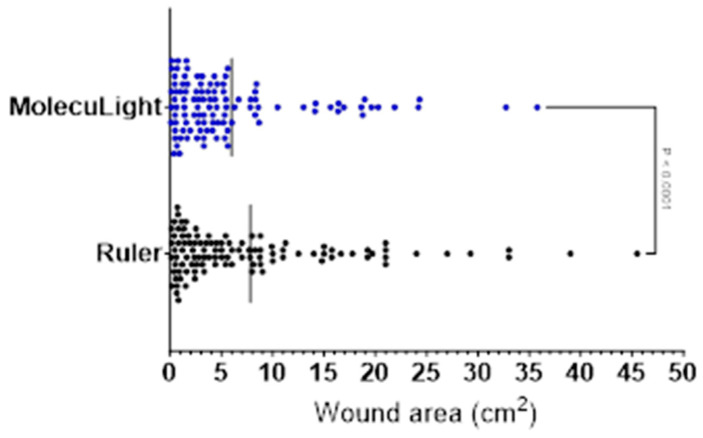
Mean difference in wound area across all visits as determined using a ruler (black) versus the digital measurement (MolecuLight^®^) software (blue).

The range of wound areas recorded was 0.10 cm^2^ to 45.50 cm^2^ for the ruler measurements and 0.07 cm^2^ to 35.76 cm^2^ for the digital MolecuLight measurements (Table 4). In 72.9% of cases (78/107), the measurement obtained by the ruler was greater than that of the MolecuLight digital wound feature. This would be considered an overestimation, due to the validated accuracy of MolecuLight wound measurement [51]. The mean difference in wound area across all visits was +1.83 cm^2^ (SD = 2.93, 95% CI [1.26, 2.39]) when determined using a ruler versus the MolecuLight digital wound measurement software (i:X Imaging 1.3); this was statistically significant (*p* < 0.0001) as per the Wilcoxon matched-pairs signed rank test (Figure 3).

When considering the average area among all measurements recorded during the study period (6.91 cm^2^, n = 214), this equates to a percentage difference of 23.34%. So, the overestimation of the ruler stands at an average of 23%. However, the average difference between the ruler versus the digital MolecuLight wound area measurements tended to decrease as the visits progressed (range = 1.41 to 2.65 cm^2^), alongside decreases in average wound area.

## 4. Discussion

The wound area reduction (WAR) during the first weeks after starting intervention on a problematic wound is a prognostic indicator of the wound’s healing outcome [37,38,39]. This study focuses on those early weeks and the findings during those assessments, with a specific focus on bacterial presence as an indicator of WAR outcomes. This study illustrates that closely and consistently monitoring and addressing the factors perpetuating these wounds while focusing on bacterial eradication during those first weeks of intervention can impact healing outcomes irrespective of wound duration at treatment onset and/or etiology. Our results show a WAR average of −46.9% (manual measurement) and −46.0% (digital measurement, MolecuLight^®^) for the entire cohort. The WAR results were not statistically significantly different between acute (−49.8% and −45.8%, respectively) and chronic wounds (−42.7% and −46.4), suggesting that this intensive approach may have a positive impact irrespective of how affected the healing process may be.

Close monitoring of a wound’s progression has been shown as an essential contributing factor to successful closure. Monitoring a wound’s progression informs treatment effectiveness, guiding educated decisions and adjustments to care plans. Regular assessments not only gauge the response to treatment and fluctuations in size but serve to flag early indicators of complications like cellulitis or osteomyelitis.

Obtaining wound measurements to track the progress of the wound is a common and effective method of assessment. However, the accuracy of the traditional manual method has been questioned recurrently. Studies have found ruler measurement overestimation to be between 29 and 43% of the wound’s area [41,42]. A systematic review looking at wound measurement procedures reported that the most accurate and reliable methods for area measurement were digital planimetry and digital imaging [40]. The digital imaging method employed in this study is reported to have an accuracy exceeding 95% [51]. Herein, when compared to the digital measurement device, manual measurements were consistently overestimated across all four assessments for the included wounds. As a result, there was no statistically significant difference in the wound area reduction (WAR) calculation between the two methods. The consistency in the overestimation of manual ruler measurements in the present study can be attributed to the fact that they were conducted by the same examiner (RD). However, inconsistencies may arise when evaluated by different examiners. Additionally, variations in individual techniques, such as observation angle and applied pressure during measurement, can introduce inconsistencies and compromise accuracy. Other limitations of the two-dimensional measurements with a flat ruler are the inaccurate representation of the irregular contours and dimensions of complex wounds. Ruler use may inadvertently include surrounding healthy tissue, leading to an overestimation of the actual wound size. This limitation is significant, particularly during specific applications like when calculating the area of a skin graft or skin substitute.

In addition to monitoring wound size changes closely, the constant evaluation of bacteria and biofilm is crucial for a positive wound healing outcome. Bacteria and biofilm are healing deterrents; they can arrest the healing cascade at the inflammatory phase, not only impeding wound closure but causing tissue damage and predisposing to infection and its complications [13,14,15,18]. A key finding of this study suggests that continued wound assessment should also aim to determine the wound’s status along the recognized bacterial contamination-to-infection continuum at the earliest time possible to obtain the best possible outcomes. A clear correlation was shown between bacterial presence at the outset and end of the study period and the WAR obtained. This is in line with other studies that have shown the predictive capabilities of bacterial fluorescence signals. Okeahialam et.al determined that fluorescence signals were an independent risk factor associated with delayed perineal wound healing [35]; similarly, Rahma et al evidenced a gradually deteriorating pattern of DFU healing, where individuals exhibiting negative bacterial fluorescence experienced more favorable outcomes followed by those with positive autofluorescence who underwent intervention, and the least favorable outcomes were observed in individuals with positive autofluorescence without any intervention [33].

**Figure 4:**
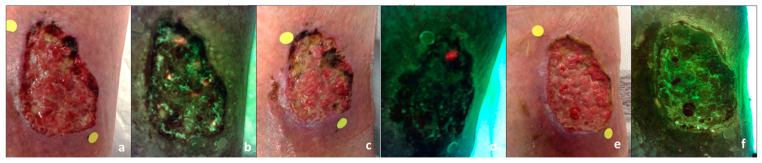
Case example of fluorescence-guided wound management. A 75-year-old male with a post-traumatic leg wound of three weeks duration. The wound had initially been sutured and dehisced. (**a**) Standard image of the wound on the lower leg at visit 1. (**b**) Corresponding fluorescence image demonstrating red fluorescence signals scattered within the wound bed. (**c**),On day 5, the standard image shows a reduction in the wound area, while the corresponding (**d**) fluorescence image shows persistent yet localized red fluorescence at 12–1 o’clock. This allows for targeted removal via debridement of that area. (**e**) By the end of the study period, the wound area was reduced from 29.25 cm^2^ to 21.87 (manual) and from 19.25 cm^2^ to 13.01 cm^2^ (digital). Therefore the WAR is 25% (manual) or 32% (digital). (**f**) Under fluorescence imaging, there is an absence of pathogenic fluorescence signals which is in line with better outcomes related to the absence of bacteria.

In the study herein, the presence of positive signals at the end of the study period was a clear marker of inferior outcomes. Within the current patient cohort, this may have resulted from the ineffectiveness of interventions in eliminating bacterial loads over the 2-week treatment period or from the de novo emergence of bacterial presence between visits 1 and 4 which remained undetected or undertreated. Figure 4 describes a case example that illustrates how fluorescence signals are used in real-time to target treatment at the bedside, modify clinical conduct, and monitor a patient’s progression. Notably, even though the progression was favorable for those wounds that were negative throughout (WAR of 49.7% per digital measurements), the cohort exhibiting the best outcomes was the group in which the positive signals were initially present and effectively eliminated throughout the course of the 2-week treatment. We hypothesize that the presence of pathogenic bacteria may play a more pivotal role in the etiology of healing arrest for some wounds (those in which it was detected by fluorescence imaging). Meanwhile, in non-healing wounds initially lacking pathogenic bacteria, other causative factors might have been more influential in hindering positive progression, as the multi-factorial origin of these types of wounds is well known. However, further studies are necessary to better elucidate this finding.

For those wounds in which fluorescence signals were found either at baseline or on subsequent visits, treatment decisions were guided by clinical assessment and expertise as well as fluorescence imaging findings. This strategy was chosen based on published studies that underscored the positive effects on healing outcomes through a shift in treatment depending on fluorescence findings at the bedside [16,33,34,36].

The timely detection and localization of pathogenic levels of bacteria and biofilm can serve to guide appropriate localized treatment whilst improving resource allocation and shortening healing times. Interventions such as debridement, antibiotics, and antimicrobial dressings should be used appropriately to prevent the wound from escalating to an established and/or complicated infection (e.g., cellulitis, osteomyelitis, amputations, gangrene, sepsis, etc.) and overall improving cost-efficiency [52].

## 5. Conclusions

This feasibility study aimed to determine the clinical utility of fluorescence imaging by understanding the correlation of WAR results to fluorescence signals. The results show that a clear relationship exists between the two, suggesting that wound assessment, TIME wound bed preparation, and treatment planning [48] can further be enhanced by technology such as fluorescence imaging. An objective, scientifically sound method to track the progression of the wound in terms of size and bacterial burden can become a much needed aid to clinicians in determining the best therapeutic approach, documenting their patients’ journeys and moving away from imprecise methods with high levels of inter and intra-reader variability.

## 6. Limitations

This study was conducted at a single site and included a relatively small heterogeneous sample in relation to age and wound etiology. Therefore, caution is needed in generalizing the results. Nonetheless, the participants were representative of the types of patients attending a dressing clinic in an acute hospital.

## Figures and Tables

**Table 1:** Cohort demographics and wound characteristics.

	Total	Acute (%)	Chronic (%)
Number of patients	26	16 (61.5)	10 (38.5)
Number of wounds	27	16 (59.3)	11 (40.7)
Age—mean (SD)	47.1 (19.5)	44.7 (19.6)	50.9 (19.7)
Gender (%)			
Male	5 (19.2)	2 (12.5)	3 (30.0)
Female	21 (80.8)	14 (87.5)	7 (70.7)
Comorbidities			
>1 *	17 (65.4)	10 (62.5)	7 (70.0)
Diabetes	6 (23.1)	3 (18.8)	3 (30.0)
COPD	1 (3.8)	0	1 (10.0)
Peripheral vascular disease	2 (7.7)	0	2 (20.0)
Kidney disease	1 (3.8)	1 (6.3)	0
Hypertension	6 (23.1)	4 (25.0)	2 (20.0)
Smoking	11 (42.3)	9 (56.3)	2 (20.0)
Wound Location (%)			
Buttocks	5 (18.5)	2 (12.5)	3 (27.3)
Abdomen	5 (18.5)	3 (18.8)	2 (18.2)
Lower leg	5 (18.5)	2 (12.5)	3 (27.3)
Thigh	2 (7.4)	2 (12.5)	0
Foot	6 (22.2)	3 18.8)	3 (27.3)
Back	2 (7.4)	2 (12.5)	0
Other	2 (7.4)	2 (12.5)	0

* Includes: Diabetes, asthma, chronic obstructive pulmonary disease (COPD), peptic ulcer disease, Crohn’s disease, ulcerative colitis, peripheral vascular disease, varicose veins, renal disease, prostate cancer, hidradenitis suppurativa, chronic lymphocytic leukemia, lymphoma, melanoma, bowel cancer, IV drug use, pancreatitis, hypertension, high cholesterol, ischemic heart disease, hypothyroidism.

**Table 2:** Wound area reduction observed among chronic versus acute wounds.

			Visit 1 Avg Wound Area (cm^2^)		Visit 4 Avg Wound Area (cm^2^)		∆ Wound Area cm^2^		∆ Wound Area %	
Type	n	%	RULER	DIGITAL *	RULER	DIGITAL *	RULER	DIGITAL *	RULER	DIGITAL *
All	27	-	9.74	7.30	5.94	4.68	−3.80	−2.62	−46.87	−46.05
Acute	16	59.3	8.85	6.11	4.56	3.57	−4.29	−2.55	−49.76	−45.80
Chronic	11	40.7	11.03	9.04	7.94	6.31	−3.09	−2.73	−42.67	−46.40

* MolecuLight^®^ i:X.

**Table 3:** Wound area reduction observed for acute and chronic wounds, subdivided by bacterial fluorescence status classification.

			Visit 1 Avg Wound Area		Visit 4 Avg Wound Area		∆ Wound Area cm^2^		∆ Wound Area %	
Classification Visit 1–Visit 4	n	%	Manual	Digital *	Manual	Digital *	Manual	Digital *	Manual	Digital *
pos-pos	5	19	20.28	17.37	16.73	13.48	−3.55	−3.89	−16.66	−22.81
pos-neg	16	59	8.11	5.45	3.46	2.44	−4.65	−3.02	−62.07	−58.63
neg-pos	2	7	4.20	4.04	4.50	3.91	0.31	−0.13	19.24	3.77
neg-neg	4	15	5.82	3.78	3.09	3.07	−2.73	−0.71	−56.89	−49.69

* MolecuLight i:X; pos-pos: fluorescence signals were positive at the outset and remained positive at end of study period; pos-neg: fluorescence signals were positive at the outset but were negative by the end of the study period; neg-pos: no fluorescence signals were evidenced at the outset, but positive fluorescence signals were present at the end of study period; neg-neg: no evidence of fluorescence either at the outset or at the end of the study period.

**Table 4:** Ruler measurement versus digital measurement (MolecuLight).

Acute and Chronic		Ruler (Wound Area in cm^2^)			Digital (Wound Area in cm^2^)			Difference (Absolute)		
Visit	N	Avg	MIN	MAX	Avg	MIN	MAX	∆ cm^2^	MIN	MAX
1	27	9.74	0.54	45.50	7.30	0.48	35.76	2.65	0.01	12.58
2	27	8.20	0.16	39.00	6.23	0.14	32.72	2.14	0.02	10.59
3	27	7.11	0.14	33.00	5.54	0.10	24.2	1.68	0.00	8.8
4	26	6.17	0.10	33.00	4.86	0.07	24.3	1.41	0.02	8.7

Avg: Average; MIN: Minimum; MAX: Maximum.

## Data Availability

The data presented in this study are available on request from the corresponding author. The data are not publicly available due to privacy.
